# Obesity and high neutrophil-to-lymphocyte ratio are prognostic factors in non-metastatic breast cancer patients

**DOI:** 10.1590/1414-431X2021e11409

**Published:** 2021-08-13

**Authors:** L.F. Orlandini, F.F. Pimentel, J.M. de Andrade, F.J.C. dos Reis, L. de Mattos-Arruda, D.G. Tiezzi

**Affiliations:** 1 Setor de Mastologia, Departamento de Ginecologia e Obstetrícia, Faculdade de Medicina de Ribeirão Preto, Universidade de São Paulo, Ribeirão Preto, SP, Brasil; 2 IrsiCaixa, Hospital Universitari Trias i Pujol, Badalona, Spain; 3 Centro de Pesquisa Avançada em Medicina, União das Faculdades dos Grandes Lagos, (UNILAGO), São José do Rio Preto, SP, Brasil

**Keywords:** Breast cancer, Obesity, Neutrophil-to-lymphocyte ratio, Prognosis, Survival

## Abstract

Obesity has been associated with an increased risk of breast cancer recurrence and death. Some readily available biomarkers associated with systemic inflammation have been receiving attention as potential prognostic indicators in cancer, including neutrophil-to-lymphocyte ratio (NLR) and platelet-to-lymphocyte ratio (PLR). This study aimed to explore the correlation between body mass index (BMI) and invasive breast cancer and the association of NLR, PLR, and BMI with breast cancer outcomes. We undertook a retrospective study to evaluate patients treated for breast cancer over 14 years. Clinicopathological data was obtained before receiving any treatment. Of the 1664 patients included with stage I-III, 567 (34%) were obese (BMI≥30 kg/m^2^). Obese patients had larger tumors compared to non-obese patients. Higher BMI was associated with recurrence and worse survival only in patients with stage I disease. NLR and PLR were classified into high and low level groups. The NLR^high^ (NLR>4) was found to be an independent prognostic factor for recurrence and mortality, while the PLR^high^ (PLR>150) group had no impact on survival. A subgroup of patients with NLR^high^ and BMI^high^ had the worst disease-free survival (P=0.046), breast cancer-specific survival (P<0.001), and overall survival (P=0.006), compared to the other groups. Patients with early-stage breast cancer bearing NLR^high^ and BMI^high^ had worse outcomes, and this might be explained by the dysfunctional milieu of obesity in adipose tissue and its effects on the immune system. This study highlights the importance of lifestyle measures and the immune system interference with clinical outcomes in the early breast cancer setting.

## Introduction

Breast cancer is the most common malignant neoplasm among women, accounting for approximately 25% of new cancer cases and 15% of cancer deaths worldwide (1). In Brazil alone, an estimated 66,280 new cases are expected to be diagnosed each year between 2020 and 2022 (2). Breast cancer is known to be a heterogeneous disease, with different clinical presentations and molecular subtypes (3).

Clinical and pathological staging, molecular profiling, and environmental factors can influence breast cancer outcomes (4). Among environmental factors, obesity has been reported as one of the most prevalent modifiable risk factors associated with chronic disease, raising awareness of its relation to cancer (5). Despite numerous studies evaluating obesity and breast cancer mortality (6), the mechanisms through which obesity exerts its effects on breast cancer survival have not been fully elucidated. Obesity seems to be particularly relevant in postmenopausal women and those with hormone receptor positive tumors (7).

Obesity and carcinogenesis have an important feature in common, which is the involvement of the inflammatory pathways (8). Dysregulated metabolism and a state of chronic subclinical inflammation, along with elevated levels of proinflammatory and immune mediators in the peripheral blood and local breast tissues, play important roles (9). There is evidence that chronic inflammation could influence tumor initiation, promotion, invasion, and metastasis (10).

Two inflammatory biomarkers recently described as prognostic factors for cancer are the peripheral blood neutrophil-to-lymphocyte ratio (NLR) and platelet-to-lymphocyte ratio (PLR) (11,12). Although their significance has not been fully elucidated, there is evidence that an inflammatory stimulus may increase the production and release of neutrophils from the bone marrow, mediated by the granulocyte colony-stimulating factor (G-CSF), interleukin (IL)-1β, IL-6, and tumor necrosis factor-α (TNF-α) (13). IL-6 also stimulates the differentiation of megakaryocytes to platelets and thrombopoietin production, thus increasing the blood platelet count (14). In contrast, neutrophilia, particularly due to TNF-α and IL-1β, has an inverse relationship with the lymphocyte count, while lymphopenia is also correlated with poor prognosis (12). NLR and PLR as biomarkers are cheap, easy to measure and monitor over time, and have been previously shown to predict worse outcomes and decreased response to treatment in some breast cancer patients (15,16).

Here, we explored the association between obesity, assessed by body mass index (BMI), and early-stage breast cancer at presentation and survival. We further investigated the prognostic significance of NLR and PLR for breast cancer recurrence and survival and the influence of BMI on these biomarkers.

## Material and Methods

### Study participants

This was a retrospective study with all patients diagnosed and treated for malignant breast neoplasms at the Breast Disease Division of Clinics Hospital of Ribeirão Preto School of Medicine, University of São Paulo (USP), between 1999 and 2013. The clinical and pathological data was obtained from the patients’ files (including paper charts and electronic medical records). The variables obtained were: age, histological type and grade, TNM stage, tumor size, positive lymph nodes, immunohistochemistry (IHC), menopausal status, weight, height, and total blood count. Treatment characteristics were also collected including surgical treatments, hormone therapy, chemotherapy, and radiotherapy. The study was approved by the local ethical committee (approval number 2.638.453/2018).

**Figure 1:**
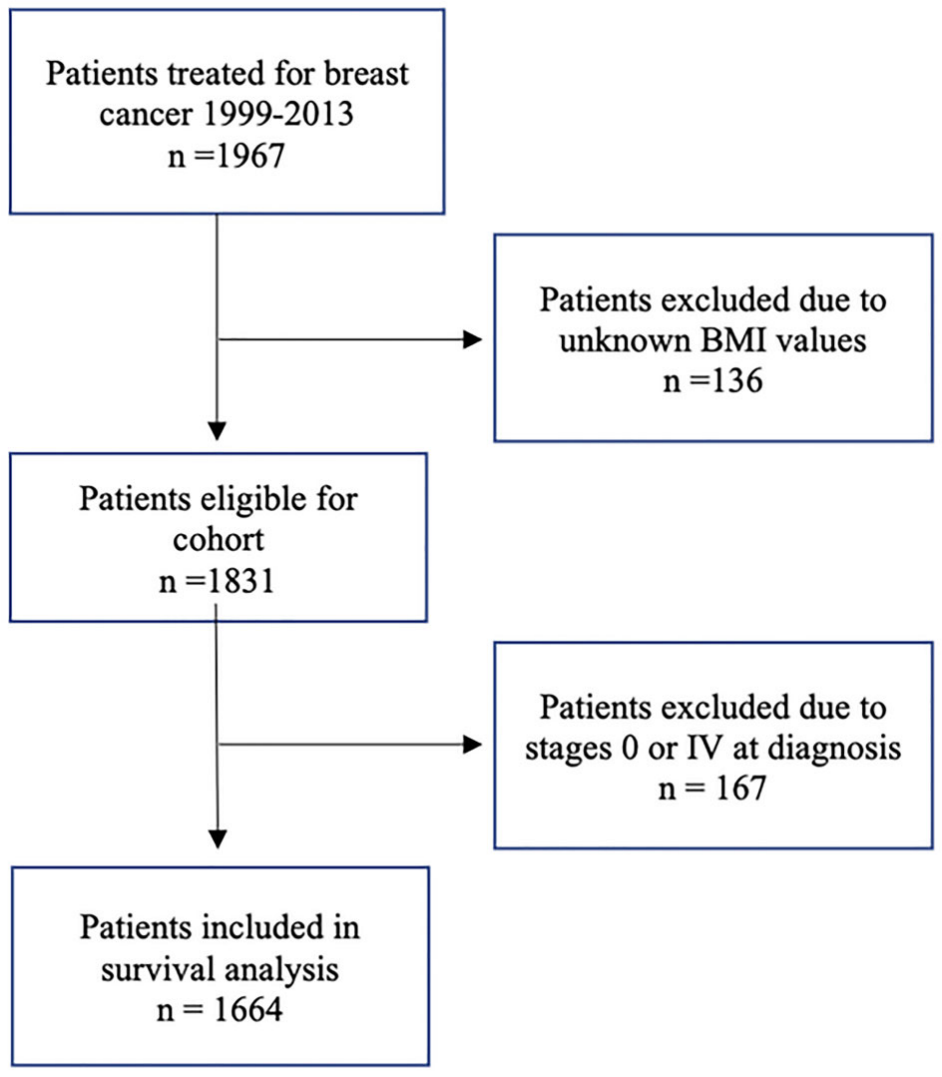
Flow diagram showing enrollment of women in the study.

Of the 1967 patients selected, 136 did not have available information about the height or weight and were not included in the study. We further restricted the cohort to those with stage I to III cancers. Other patients with missing variables were also excluded from the analyses; however, none of them had more than 10% of missing data. Data were collected from the date of diagnosis until death or until July 1, 2018, which was chosen as the final date of observation. In case of loss to follow-up, the time was censored at the date of the last information in the medical records. Disease-free survival (DFS) was defined as the time from the diagnosis to the development of locoregional or contralateral recurrence or evidence of distant metastasis. Breast cancer-specific survival (BCSS) was defined as the duration from the date of diagnosis until death due to breast cancer and overall survival (OS) was defined as the duration from the date of diagnosis to death, with no restriction on the cause of death. Patients with unknown menopausal status (n=129) were considered to be postmenopausal if they were ≥51 years old. This assumption was based on existing studies conducted in the same region (17) (Figure 1).

### Tumor subtypes and BMI

The amplification/overexpression of human epidermal growth factor receptor 2 (HER2) and the expression of hormonal receptors were determined by IHC in accordance with specific guidelines (18,19). HER2 positivity was established in accordance with the pathology report and the protocols followed at the time of diagnosis, as recorded in the clinical chart. Fluorescent *in situ* hybridization (FISH) was used in patients with HER2 2+ IHC results. The subtype was considered to be luminal-like if the estrogen receptor (ER) and/or the progesterone receptor (PR) were positive and HER2 was negative; luminal/HER2-like if ER and/or PR were positive and HER2 was positive; HER2-like if ER and PR were negative and HER2 was positive; and triple negative (TN) when ER, PR, and HER2 were negative. For staging, we used the American Joint Committee on Cancer Staging Manual, 7th edition (20).

Height and weight data were collected prior to the first cancer treatment. BMI was calculated as weight (in kilograms) divided by height (in meters) squared, and obesity was defined as BMI≥30 kg/m^2^ (21). BMI was initially explored as a continuous variable, but as there was no difference in the results, we chose to analyze it as a categorical variable, consistent with the majority of published studies.

### Laboratory investigations

Hematologic samples collected prior to the first cancer treatment were considered in the analyses. Absolute blood counts of different cell types were determined. The ratios of NLR, which is the absolute number of neutrophils divided by the absolute number of lymphocytes, and PLR, which is the absolute value of platelets divided by the absolute value of lymphocytes, were calculated (11). We used receiver operating characteristic (ROC) curve analyses to estimate the best cut-off values for both parameters.

### Statistical analysis

To evaluate the differences between the groups, we used the chi-squared test for categorical variables and the Mann-Whitney or Kuskall-Wallis tests for continuous variables. Normality was tested with the Shapiro-Wilk test. We used Cox multivariate regression model for analyzing variables considered significant (P<0.05) with univariate analyses and BMI.

Kaplan-Meier curves were used to estimate cancer recurrence, BCSS, and OS at 10 years, and the survival differences between the groups were tested by the log-rank test. We set the level of significance at 0.05 and conducted all analyses using the R software version 3.6.1 (R Core Team, Austria).

## Results

### Population characteristics

Overall, 1664 early breast cancer patients were included in the study, of which 567 (34%) had a BMI≥30 kg/m^2^ at the time of diagnosis. Obese patients were older (P=0.03) and had a larger median tumor size (median 22 *vs* 20 mm, P=0.01) compared to non-obese patients. There were no significant differences between obese and non-obese women in terms of the histologic type and grade, cancer staging, hormone receptors, and HER2. With regard to the treatment variables, obese patients were slightly more likely to receive radiotherapy during the course of the treatment (P=0.03), but with no difference in type of surgery or stage. Table 1 describes the cohort characteristics according to the BMI.

**Table 1:** Baseline characteristics of all patients with breast cancer (n=1664), stratified by the body mass index (BMI).

Characteristics	BMI≥30 kg/m^2^ (n=567)	BMI<30 kg/m^2^ (n=1097)	P value
BMI, average (SD)	35.0 (4.5)	25.1 (3.1)	**0.01**
Age (years), median (range)	56 (25.0-89.0)	54.2 (23.6-93.8)	**0.03**
Tumor size (mm), median (range)	22 (0-154)	20 (0-140)	**0.01**
	n (%)	n (%)	
Postmenopausal status	370 (65)	662 (60)	0.05
Histology			
Ductal	506 (89)	967 (88)	0.46
Lobular	25 (4)	45 (4)	
Other	31 (6)	77 (7)	
Unknown	5 (1)	8 (1)	
Grade^a^			
1	118 (21)	224 (20)	0.96
2	304 (54)	585 (53)	
3	132 (23)	246 (22)	
Unknown	13 (2)	42 (4)	
Stage (TNM)			
I (A+B)	110 (20)	215 (20)	0.25
II (A+B)	228 (40)	482 (44)	
III (A+B+C)	229 (40)	400 (36)	
Subtype			
Luminal-like	351 (62)	640 (59)	0.32
Luminal/HER2-like	77 (14)	157 (15)	
HER2-like	45 (8)	115 (10)	
Triple negative	85 (15)	169 (15)	
Unknown	9 (1)	16 (1)	
Estrogen receptor status			
Positive	423 (75)	789 (73)	0.25
Negative	138 (24)	297 (26)	
Unknown	6 (1)	11 (1)	
Progesterone receptor status			
Positive	360 (64)	645 (59)	0.07
Negative	201 (35)	440 (40)	
Unknown	6 (1)	12 (1)	
HER2 status			
Positive	122 (22)	272 (25)	0.15
Negative	437 (77)	809 (74)	
Unknown	8 (1)	16 (1)	
NLR median (range)	1.93 (0.23-15.17)	1.99 (0.18-31.67)	0.67
PLR median (range)	122.61 (26.35-875)	129.36 (15.29-655)	0.40
First treatment			
Upfront surgery	352 (56)	697 (58)	0.58
Neoadjuvant chemotherapy	243 (39)	459 (38)	
NEO endocrine therapy	25 (4)	46 (4)	
NEO radiotherapy	0 (0)	5 (0)	
Treatment			
Surgery approach			
Conservative surgery	312 (56)	577 (54)	0.34
Mastectomy	240 (44)	493 (46)	
Axillary approach			
Sentinel node biopsy	181 (34)	325 (32)	0.36
Axillary node dissection	369 (66)	738 (68)	
Chemotherapy	401 (68)	792 (68)	0.93
Endocrine therapy	411 (73)	751 (68)	0.08
Radiotherapy	433 (78)	783 (73)	**0.03**
Trastuzumab	57 (10)	121 (11)	0.56

a Nottingham histologic score system; BMI: body mass index; NLR: neutrophil-to-lymphocyte ratio; PLR: platelet-to-lymphocyte ratio; NEO: neoadjuvant. Bold type indicates statistical significance (chi-squared test for categorical variables and Kuskall-Wallis test for continuous variables).

### Association between BMI, NLR, and PLR

The median pre-treatment NLR and PLR were 1.93 and 122.6 for obese patients and 1.99 and 129.3 for non-obese patients, respectively. ROC curves did not identify an ideal cut-off point for NLR and PLR (AUC=0.53 and 0.51, respectively). Hence, we decided to use NLR>4 (NLR^high^) and PLR >150 (PLR^high^) as cut-off, since these values demonstrated statistical significance and were chosen in previously published studies (22,23).

On univariate analysis, NLR greater than 4 had an impact on DFS, BCSS, and OS only in obese patients, while a higher PLR showed an influence on DFS and BCSS, but not on OS in obese. Non-obese patients were not impacted by high NLR or PLR (Supplementary Table S1).

### Recurrence and survival analysis

The median follow-up time was 6.7 years in obese patients and 6.9 years in non-obese patients (P=0.91). During the study period, 482 patients experienced a locoregional or distant recurrences; the proportion was 26.3% in non-obese patients and 26.5% in obese patients (P=0.93). In the total population, there were 409 deaths due to breast cancer, and 531 deaths due to all causes, with no differences between the two groups (P=0.75) (Supplementary Table S2).

On performing multivariate analysis, including BMI and all variables with statistical significance on univariate analysis, BMI was not found to be a significant predictor of cancer recurrence, BCSS, and OS. The main independent predictors of worse outcomes were grade, stage III, HER2, TN subtypes, and NLR^high^. The PLR was not statistically significant on multivariate analysis and was not considered an independent predictor of worse outcomes (Supplementary Table S3). When patients were stratified by menopausal status, we found that BMI was not a predictor of more aggressive tumors or worse outcomes in the population of postmenopausal women on multivariate analysis (Supplementary Table S4).

**Figure 2:**
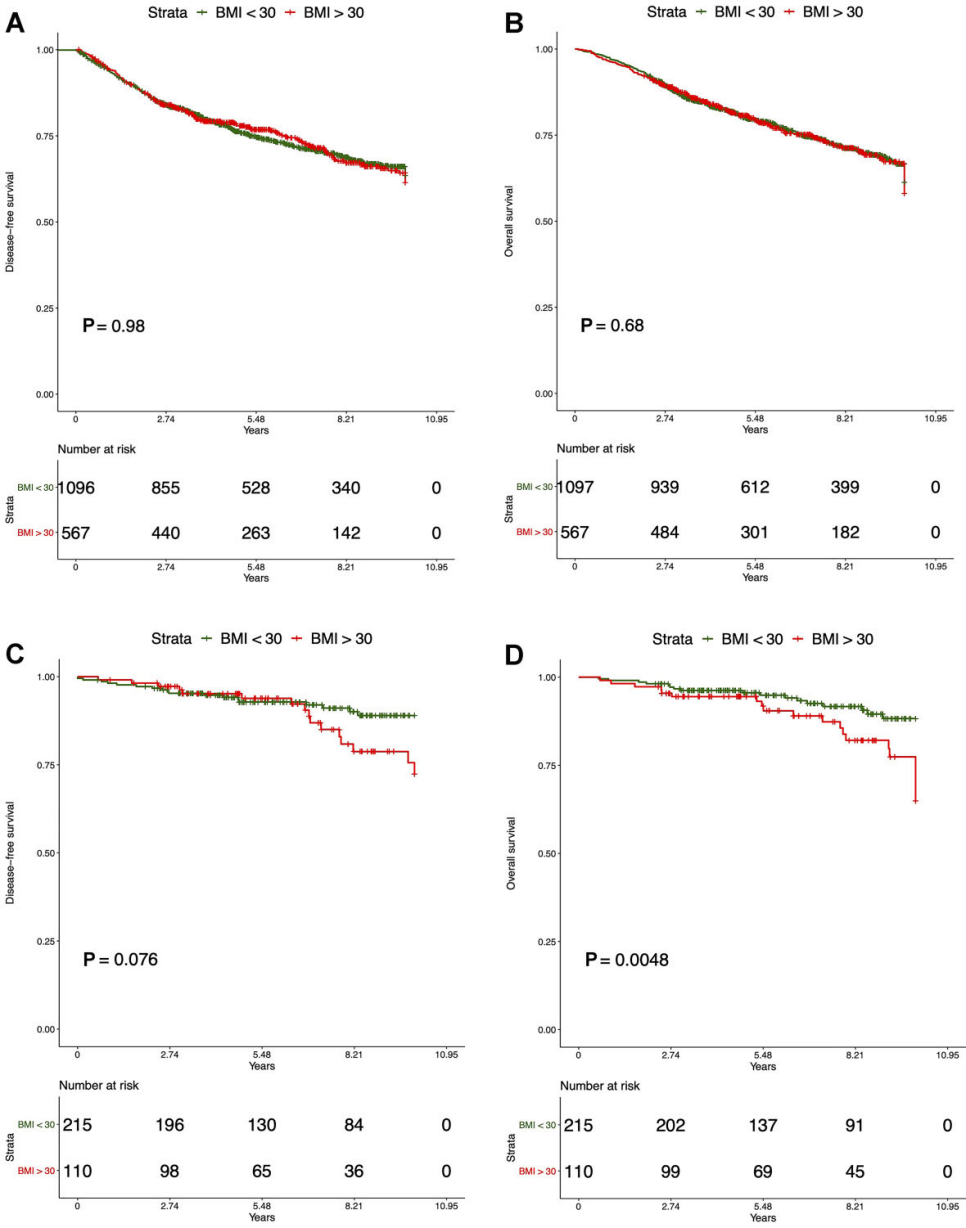
Survival analysis of stage I to III breast cancer patients, according to the body mass index (BMI). **A**, Disease-free survival in all patients; **B**, overall survival in all patients; **C**, disease-free survival in stage I patients at diagnosis; **D**, overall survival in stage I patients at diagnosis.

**Figure 3:**
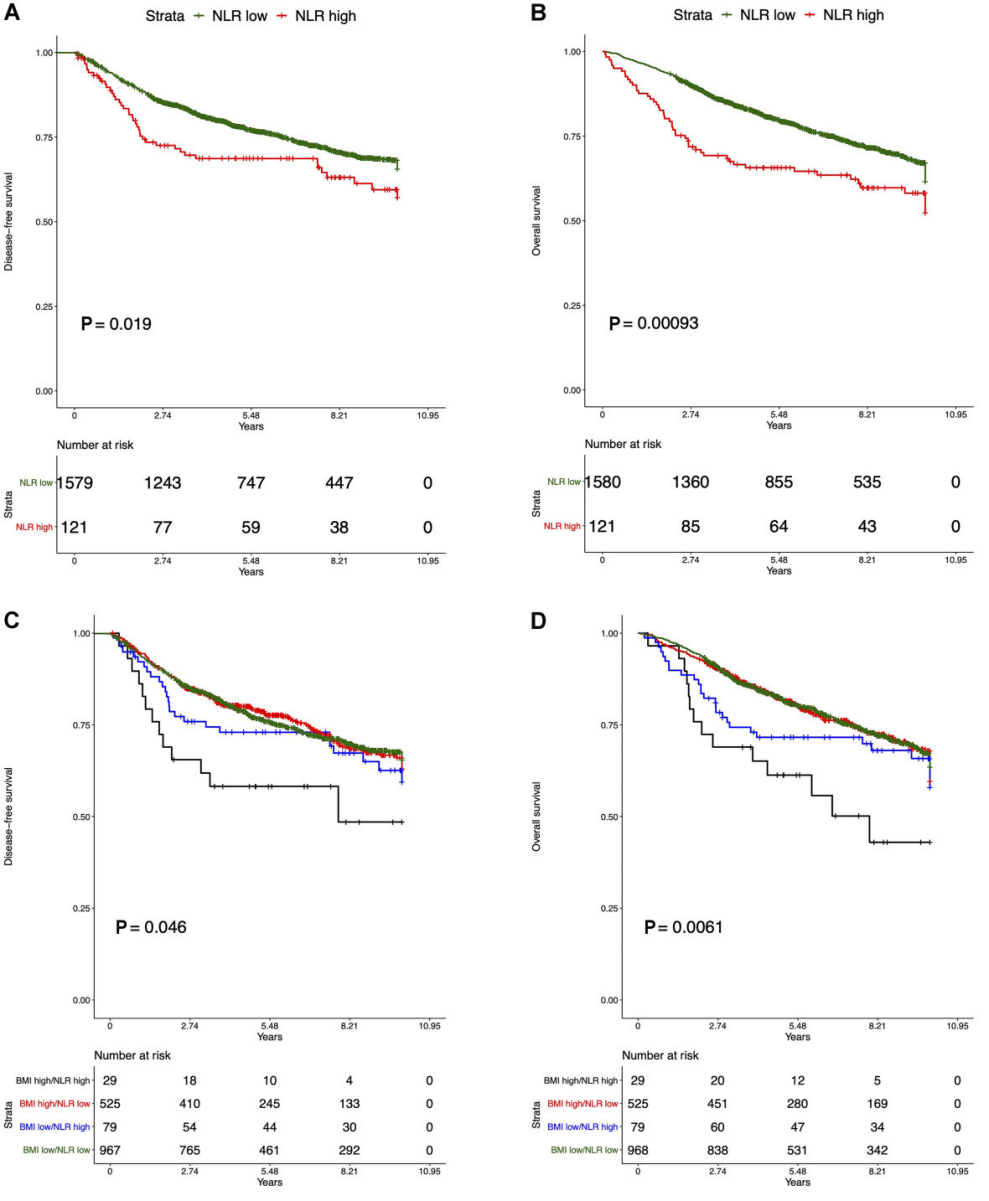
Survival analysis of stage I to III breast cancer patients, according to the neutrophil-to-lymphocyte ratio (NLR). **A**, Disease-free survival; **B**, overall survival. Curves (**C** and **D**) show disease-free survival and overall-survival for combined groups of NLR and body mass index (BMI), respectively. The black line indicates the patients with both NLR^high^ (>4) and BMI^high^ (≥30 kg/m^2^), who had the worst outcomes.

Kaplan-Meier curves with log-rank tests for recurrence and overall survival at ten years showed that BMI did not influence outcomes in the total population (Figure 2A and B). However, when stratified by stage, we observed a negative impact of obesity only in patients with stage I disease, where it was associated with worse OS (log-rank test P=0.0048) and slightly shorter, but not significant, DFS (log-rank test P=0.076) (Figure 2C and D). In more advanced breast cancer stages, we did not find any significant correlation. Additionally, Figure 3A and B showed recurrence and survival curves of NLR^high^ compared to NLR^low^ in total population (log-rank P<0.001).

On examining the correlation of NLR and BMI with the outcomes, we observed that a subgroup of breast cancer patients with NLR^high^ and BMI ≥30 kg/m^2^ (BMI^high^) had a significantly lower DFS (P=0.046, log-rank) and OS (P=0.006, log-rank) compared to the other groups (Figure 3C and D).

## Discussion

In this study, we explored the association between BMI and inflammatory biomarkers in a large cohort of patients with invasive breast cancer. Here we showed that patients with non-metastatic breast cancer bearing NLR^high^ and BMI^high^ had worse outcomes. NLR^high^ status at diagnosis was observed to be an independent prognostic factor associated with a shorter DFS and worse survival, particularly in the subset of patients with a NLR^high^ and obesity.

Some recent meta-analyses have evaluated the association between obesity and breast cancer. Chen et al. (24) analyzed 31 studies with more than three million subjects and described an increased risk of postmenopausal breast cancer among obese women (RR: 1.33 (95%CI: 1.20-1.48). Chan et al. (6) evaluated four BMI categories among 213,075 patients and concluded that the relative risk in obese versus non-obese patients was 1.41 (95%CI: 1.29-1.53) for all-cause mortality and 1.35 (95%CI: 1.24-1.47) for BCSS. Protani et al. (25) pooled 43 studies and showed that obese patients had worse outcomes than non-obese patients, with a hazard ratio (HR) of 1.33 (95%CI: 1.21-1.47) for OS and 1.33 (95%CI: 1.19-1.50) for BCSS.

These meta-analyses were based mostly on studies conducted in high-income countries with established and structured screening programs and widespread access to healthcare services, as demonstrated by the fact that up to 80% of these patients were diagnosed in early-stages of breast cancer (26).

To our knowledge, our study is the first to explore the association between BMI and inflammatory biomarkers in the prognosis of Brazilian breast cancer patients. In Brazil, where more than 20% of the women are considered to be obese (27), approximately 40% of breast cancer cases are diagnosed at locally advanced or metastatic stages (28). This seems to be related to the absence of a well-structured population-based screening program as well as difficulties in accessing healthcare services (29). Our findings were also consistent with a previous study by Moore et al. (30) that found that obesity had a negative impact only in early-stage breast cancer patients. This suggests that obesity has a negative impact on a group of women with favorable prognostic features, since the modest effect of obesity may be mitigated by the worse prognosis and the amount of therapies used in the more advanced stages (7,30).

We did find larger tumors among the obese patients, which may be due to the fact that obese women often have larger breasts with less palpable masses (31). These women may also be less likely to participate in breast cancer screening programs, due to low self-esteem and poor body image (32). In terms of age and menopause, it is well known that women tend to gain weight after menopause, and that the breast cancer risk increases with age (33).

In this study, it was observed that PLR, despite having a negative association with recurrence and BCSS, was not an independent prognostic factor in multivariate analysis. This finding was in agreement with a previous study by Azab et al. (34) that demonstrated that NLR was superior to PLR as a prognostic factor for worse outcomes in breast cancer. We did not find an association between these biomarkers and breast cancer subtypes, although three recent meta-analyses have demonstrated that these biomarkers are more commonly associated with HER2 and TN breast cancers (11,12,35). Although there is increasing evidence that these ratios, when obtained prior to treatment, can act as prognostic biomarkers of breast cancer, the cut-off values have not yet been established. In a recent meta-analysis with 8,563 breast cancer patients, it was demonstrated that a higher NLR was associated with worse OS (HR: 2.56; 95%CI: 1.96-3.35; P<0.001) and DFS (HR: 1.74; 95%CI: 1.47-2.07; P<0.001). The cut-off values for a high NLR ranged from 1.9 to 5.0 in the 15 studies included (36). In line with our study, some previous studies have used a cut-off value of 4.0 for NLR (23,36). Similarly, several cut-off values have been used for PLR, but no value has been established (12,15).

Some previous studies have suggested that despite the increase in the number of both neutrophils and lymphocytes with weight gain, the NLR remains stable regardless of the BMI category, and could be less influenced by other physiological and pathological factors (34,37,38).

In our study, we found that a subset of patients with a high NLR and high BMI had the shortest DFS and worse survival. These findings may be explained by the obesity-associated inflammation and its effects on the immune response. A recent review described the mechanisms whereby chronic adipose tissue inflammation, with altered levels of adipokines and upregulation of cytokines (IL-1β, IL-6, and TNF-α), plays an important role in breast cancer prognosis (10). In addition, obese women are known to have more frequent surgical complications, chemotherapy under-dosing, and more baseline comorbidities (9).

This study has some limitations. Although BMI is typically used in retrospective studies, it may not be ideal to measure obesity in elderly women, who are the primary age group at risk of breast cancer. This is because with menopause and advancing age, women lose height, bone mass, and muscle mass, as well as accumulate visceral fat (39), all factors that are not measured by BMI. Moreover, studies have shown that sarcopenia and muscle mass can be underestimated by BMI and can be important predictors of breast cancer mortality, even in the setting of a normal BMI (40). Another limitation is that we could not evaluate the lifetime use of hormonal therapy because these data were not available. In addition, due to the inherent limitations of observational studies, we were unable to explore potentially interesting factors such as lifestyle, abdominal and hip circumference, physical activity, smoking status, and diet. Further studies are required to explore the role of obesity in breast cancer with more accurate measures of body fat and body mass. Thus, prospective studies are needed to validate standard cut-off values defining high PLR and NLR.

In conclusion, patients with non-metastatic breast cancer bearing NLR^high^ and BMI^high^ had worse outcomes, and this might be explained by the obesity-associated inflammation in adipose tissue and its effects on the immune system. NLR^high^, but not PLR^high^, was an independent prognostic factor for worse breast cancer recurrence and survival, particularly, in a subgroup of patients with high NLR and obesity. This study highlights the importance of lifestyle measures and the immune system interference with outcomes in the early breast cancer setting.

## Supplementary Material

Click here to view [pdf].
